# Could oral hygiene prevent cases of at-home-acquired Legionnaires’ disease? – Results of a comprehensive case–control study on infection sources, risk, and protective behaviors

**DOI:** 10.3389/fmicb.2023.1199572

**Published:** 2023-06-15

**Authors:** Ann-Sophie Lehfeld, Franziska Reber, Marina M. Lewandowsky, Heiko J. Jahn, Christian Lück, Markus Petzold, Benedikt Schaefer, Anna-Rachel Germelmann, Katrin Lorenz, Udo Buchholz

**Affiliations:** ^1^Department of Infectious Disease Epidemiology, Robert Koch Institute, Berlin, Germany; ^2^Faculty of Medicine Carl Gustav Carus, Institute of Medical Microbiology and Virology, National Consulting Laboratory for Legionella, Technische Universität Dresden, Dresden, Germany; ^3^Section II 3.5 Microbiology of Drinking Water and Swimming Pool Water, German Environment Agency, Bad Elster, Germany; ^4^Independent Researcher, Berlin, Germany; ^5^Department of Periodontology, Technische Universität Dresden, Dresden, Germany

**Keywords:** Legionnaires’ disease, *Legionella*, dentures, oral hygiene, LeTriWa study

## Abstract

**Introduction:**

The “LeTriWa study” on community-acquired cases of Legionnaires’ disease (LD) found that most cases likely acquired their infection at home (AHALD). However, which sources confer the infection is largely unknown. We therefore analyzed the data set from the LeTriWa study to find out if individual sources were associated with AHALD and if specific behavioral habits may increase or lower the risk for AHALD.

**Methods:**

During the study we had used two comparison groups: (i) controls matched for age group and hospital (“controls”), (ii) household members of cases with AHALD (“AHALD-HHM”). We inquired about exposure to water sources, such as showering or wearing dentures, as well as behavioral factors and habits related to oral hygiene. We took standardized household bathroom water and biofilm samples of both cases with AHALD and controls, and in addition from households of cases with AHALD only samples from suspect residential (non-)drinking water sources. We first conducted bivariate analyses for infection sources and behaviors, followed by multivariable analyses.

**Results:**

There were 124 cases with AHALD, 217 controls and 59 AHALD-HHM. In bivariate analyses using controls for comparison, wearing dentures was the only variable significantly positively associated (odds ratio (OR) = 1.7, 95% confidence interval (CI) = 1.1–2.7, *p*-value = 0.02). Behavioral factors such as showering, letting water run before use and not being alcohol abstinent were significantly negatively associated, smoking was significantly positively associated. In a multivariable analysis, we identified good oral hygiene as a preventive factor for both denture wearers (OR = 0.33, 95% CI = 0.13–0.83, *p*-value = 0.02) and non-denture wearers (OR = 0.32, 95% CI = 0.10–1.04, *p*-value = 0.06). Analyses of comparisons with AHALD-HHM showed similar effects but lacked statistical power. We identified *Legionella* in 16 residential (non-)drinking water sources, one of which was a PCR-positive scratch sample of dentures.

**Discussion:**

Wearing (inadequately cleaned) dentures or poor oral hygiene might confer an increased risk for AHALD, and oral hygiene may prevent AHALD. The hypothesis that *Legionella* in oral biofilm or dental plaque may be the cause of cases with AHALD should be examined further. If confirmed this may open new and simple avenues for the prevention of LD.

## 1. Introduction

Legionnaires’ disease (LD) is a type of pneumonia that is caused by bacteria of the genus *Legionella*, mostly by the species *Legionella (L.) pneumophila* with a preponderance to serogroup 1 and monoclonal antibody (MAb) type 3/1 [Dresden panel (Helbig et al., 2012)]. In Germany, *Legionella* infections are reportable to the public health system. On epidemiological grounds three categories are distinguished: travel-associated LD (approximately 20%), hospital-associated LD (5–10%) and community-acquired LD (CALD; 70–75%) (Brodhun and Buchholz, 2021). This categorization is, however, quite rough and only uses the information where a case has stayed in the likely period of infection. It does not give any information in regards to the exact source. For cases of CALD a recent report by the National Academy of Sciences, Engineering and Medicine (NASEM) stated that “[…] it is largely unknown how often private water sources, particularly in individual homes, are the environmental exposure source for sporadic cases.” (The National Academy of Sciences of Engineering and Medicine, 2019). Studies have attempted to identify infection sources for cases with CALD but were successful in not more than 5–10% (Campese et al., 2011; Den Boer et al., 2015). From 2016–2020 we conducted a study in the city of Berlin and published preliminary analyses [LeTriWa study; (Buchholz et al., 2020)]. In the meantime, results of the final data are published (Lehfeld et al., 2022). Combining several methods, we were able to attribute evidence-based infection source types to about half of cases with CALD. We distinguished three types of infection sources: (a) sources outside of the cases’ residence (external sources, such as shower at a swimming pool; 16%), (b) residential non-drinking water sources, such as watering can or wearing dentures (6%), and (c) residential drinking water (27%). Because we had made great efforts to identify external sources, we felt that it is justified to assume that the remaining 51% did not originate from external sources, but rather also from a residential source. Thus, a total of 84% of cases may have acquired their infection in their residence (Lehfeld et al., 2022). From here on we will call this portion “at-home-acquired Legionnaires’ disease” (AHALD).

Which sources exactly are transmitting legionellae and subsequently lead to LD at home is largely unknown. While showers are frequently assumed as the main source of infection in indoor settings little evidence has accumulated to back this up (Breiman et al., 1990; Silk et al., 2013). In the LeTriWa study we found evidence that not all strains of the genus *Legionella* or the species *L. pneumophila* are equally pathogenic. The degree of *Legionella* concentration found in water samples was not associated with disease. A clear association of cases of CALD with the presence of MAb 3/1-positive *Legionella* in the drinking water (Buchholz et al., 2020) suggested that even small numbers of *Legionella* may lead to disease and perhaps from unexpected sources.

Thus, while cases with AHALD likely form a substantial portion of cases with CALD little is known about the risk factors associated, or conversely, how AHALD can effectively be prevented. We analyzed the data set from the LeTriWa study with the following goals:

(1) Can individual sources be associated with AHALD?
(2) Is there potential for preventive behavior lowering the risk for AHALD?

## 2. Materials and methods

The setup of the LeTriWa study and preliminary results have been reported in detail elsewhere (Buchholz et al., 2020). Briefly, between December 2016 and August 2020 cases of CALD in Berlin were recruited to take part in a case control study with the aim to identify sources for each individual’s infection as well as to determine risk factors for CALD by comparing exposure frequencies among cases and hospital/district- and age group matched controls through calculation of odds ratios (OR). We intended to recruit two controls for each case. We conducted a questionnaire inquiring about demographic factors, educational level, underlying conditions, exposure to a number of potential sources for CALD as well as habits potentially related to CALD. We elicited consumption of alcohol using the AUDIT-C questions (Bush et al., 1998) and categorized it into never-drinkers, moderate drinkers and risk drinkers (Gual et al., 2002). Few variables were added after the field phase had already started. Sources in question to cause CALD were for example showers, humidifiers, devices used by persons with sleep apnea (continuous positive airway pressure (CPAP) equipment), indoor artificial decorative fountains and potting soil. Habits potentially related to CALD included for example the frequency and duration of using the shower, staying in the bathroom while the bathtub is being filled, and wearing dentures. We took standardized household water and biofilm samples from the bathroom of both cases and controls as described in Buchholz et al. (2020). In addition, when we elicited a suspect source, e.g., a water filter, that a case had been exposed to we took (water and, if possible, biofilm) samples from these sources, too. We added sampling of dentures later in the study. A schematic overview of the data collection is shown in Supplementary Figure S1.

Apart from controls we also asked household members (HHM) of cases to answer to the questionnaire. HHM served as a second comparison group.

For the analysis presented here, we used data from all cases with AHALD and compared their exposure to possible infection sources with that of controls, as well as that of AHALD-HHM. To broaden the information and degree of exposure to infection sources with likely universal or close to universal use, such as using a shower or using water from the tap, we also asked about behaviors/habits related to these sources. For example: we asked about the weekly frequency of having a shower, if showers were taken in the morning, midday or evening, and if the person was the first person to use a shower after the residence had been left vacant (for more than 24 h). In addition, we frequently asked not only whether a behavior was prevalent, but also about its frequency, e.g., how many times teeth were brushed per week.

We **first** conducted bivariate analyses to calculate the odds ratio for exposure, separately for each comparison group. For the test statistic we used Chi-square or – when the number of observations was less than five in any of the cells analyzed – Fisher exact statistics. For categorical variables we calculated the value of p for trend using the Mantel–Haenszel test for trend. Because wearing dentures was significantly negatively associated with AHALD we separated cases, controls and AHALD-HHM in two groups: those wearing dentures and those who did not. In a **second** step we analyzed in bivariate analysis only variables related to the cleaning of dentures for the first group and only variables related to oral hygiene for the second group, including the variable “not being abstinent to alcohol” for both groups. In a **third** step we performed multivariable logistic regression analyses for both groups using STATA (v17; College Station, TX, United States). We wished to include the variables “no alcohol abstinence,” sex, age group, education level and smoking in the model, as these were considered potential confounding factors. Because finer age strata and educational levels led to instable models we used two categories for each variable (age: ≤74 years, >74 years; educational level: graduation from high school or university versus anything lower than that). OR below 1 indicate a negative (“preventive”) association, OR above 1 indicate a positive (“predictive”) association.

## 3. Results

Our study population consisted of 124 cases with AHALD, 217 controls and 59 (66% of 89 potentially recruitable (aged 18+ years)) HHM of cases with AHALD. The control-to-case ratio was 1.75: 1. Among cases with AHALD 66% were male and 34% were female, 9% were less than 50 years old, 48% were 50–74 years old and 43% were older than 74 years (Table 1). Controls did not differ from cases with AHALD by age group and sex, but were significantly more likely to have reached a higher educational level (graduated from high school or had a college degree). Household members of case-patients with AHALD did not differ in age group and educational level, but were significantly more frequently female.

**Table 1:** Study population.

	Cases with AHALD			Controls						AHALD-HHM					
	*N*	*n*	%	*N*	*n*	%	OR	95% CI	*p*	*N*	*n*	%	OR	95% CI	*p*
Gender															
Female	124	42	34	217	85	39	ref			59	40	68	ref		
Male	124	82	66	217	132	61	1.3	0.8–2.1	0.33	59	19	32	4.1	2.0–8.4	<0.001
Age group															
<50 years	124	11	9	217	21	10	ref			59	4	7	ref		
50–74 years	124	60	48	217	126	58	1.0	0.4–2.1	0.91	59	31	53	0.7	0.2–2.4	0.57
>74 years	124	53	43	217	70	32	1.4	0.6–3.1	0.46	59	24	41	0.8	0.3–2.8	0.73
Educational level															
Leaving school after 9th grade	124	49	40	216	52	24	ref			58	17	29	ref		
Graduated from secondary school	124	40	32	216	69	32	0.6	0.4–1.1	0.08	58	21	36	0.7	0.3–1.4	0.29
High school or university graduation	124	35	28	216	95	44	0.4	0.2–0.7	0.001	58	20	34	0.6	0.3–1.3	0.21

Cases of Legionnaires’ disease who likely or presumably acquired infection at home (AHALD) and two comparison groups: controls and household members of case-patients with AHALD (AHALD-HHM); LeTriWa study, Berlin, Germany, 2016–2020.

OR, Odds ratio; CI, confidence interval; *p*, value of *p*; ref, reference.

### 3.1. Microbiological results

Among cases with AHALD 60 (50%) of 119 showers and 64 (53%) of 121 bathroom faucets, respectively, contained *Legionella* spp. Among controls 95 (50%) of 190 showers and 94 (49%) of 191 bathroom faucets contained *Legionella* spp. respectively. Among cases 20 (17%) showers and 23 (19%) bathroom faucets contained MAb 3/1-positive *Legionella* compared with 9 (5%) showers and 5 (3%) bathroom faucets among controls, respectively (Table 2). This resulted in an OR of 1.0 (95% confidence interval (CI) = 0.6–1.7, value of *p* = 0.94) for *Legionella* spp. in showers, 1.2 (95% CI = 0.7–1.9, value of *p* = 0.53) for *Legionella* spp. in bathroom faucets, but 4.1 (95% CI = 1.7–10.5, value of *p* < 0.001) for MAb 3/1-positive *Legionella* in showers and 8.7 (95% CI = 3.1–30.1, value of *p* < 0.001) for MAb 3/1-positive *Legionella* in bathroom faucets. Table 2 shows further results of sampled residential drinking water or residential non-drinking water sources of cases with AHALD where we identified *Legionella*. We found legionellae in any of 16 drinking water or non-drinking water sources in the household (including standard household samples), and MAb 3/1-positive *Legionella* were found in 9 (50%) of these 16 *Legionella*-positive sources (Table 2). We identified legionellae from one (9%) of 11 scratch samples from dentures and from one (100%) of 1 sample where a biofilm sample of the denture was diluted in the water that was used for soaking the denture.

**Table 2:** Frequency and proportion of *Legionella* and monoclonal antibody (MAb) 3/1-positive *Legionella* in standardized household water and biofilm samples from the bathroom among cases who likely or presumably acquired Legionnaires’ disease at home (AHALD) and controls, as well as in other residential drinking or residential non-drinking water sources where at least one contained *Legionella*.

Potential source	Type of infection source	Number of sampled sources	Number typable	Number with *Legionella*	Proportion with *Legionella*	Number with MAb 3/1-positive *Legionella*	Proportion with MAb 3/1-positive *Legionella*
**Standardized household water and biofilm samples**							
**Cases with AHALD**							
Shower	RDW*	121	119	60	50%	20	17%
Bathroom faucet	RDW	121	121	64	53%	23	19%
**Controls**							
Shower	RDW	191	190	95	50%	9	5%
Bathroom faucet	RDW	191	191	94	49%	5	3%
Cases with AHALD: other, non-standardized samples (sources types where at least one source contained Legionella**)							
Kitchen faucet	RDW	37	37	17	46%	5	14%
Faucet of another bathroom	RDW	26	26	13	50%	2	8%
Other shower/bathtub	RDW	18	18	6	33%	2	11%
Other RDW sources	RDW	7	6	2	33%	0	0%
Dentures	RnDW*	11	9	1	11%	0	0%
Biofilm sample of denture diluted in the water used to soak the denture	RnDW	1	1	1	100%	0	0%
Water container***	RnDW	15	12	4	33%	1	8%
Water sprayer	RnDW	8	8	2	25%	0	0%
Room humidifier	RnDW	4	4	3	75%	1	25%
Toilet	RnDW	7	4	1	25%	0	0%
Sparkling water maker	RnDW	3	3	1	33%	0	0%
Inhalation device	RnDW	3	3	1	33%	0	0%
Water filter	RnDW	3	2	1	50%	1	50%
Potting soil	RnDW	1	1	1	100%	1	100%

Residential drinking or residential non-drinking water sources were only selectively taken from households of cases with AHALD; *N* = 122 cases with AHALD, for two cases with AHALD no standardized household water and biofilm samples could be collected; LeTriWa study, Berlin, Germany, 2016–2020. * RDW, residential drinking water; RnDW, residential non-drinking water, ** *Legionella* negative sources or sources where it could not be determined if they contained *Legionella* were: siphon of the bathroom basin, aquarium, night guard, steam cleaner, previously used shower head, ice cubes, garden devices (such as water sprinkler, water hose, pond, well), oral irrigator, swimming pool, decorative fountain, cat drinking fountain, samples of the water used for soaking the denture, *** includes, e.g., watering can, bird bath, and flower vase.

### 3.2. Analytical results of potential sources, behavior, knowledge and oral hygiene

**Figure 1:**
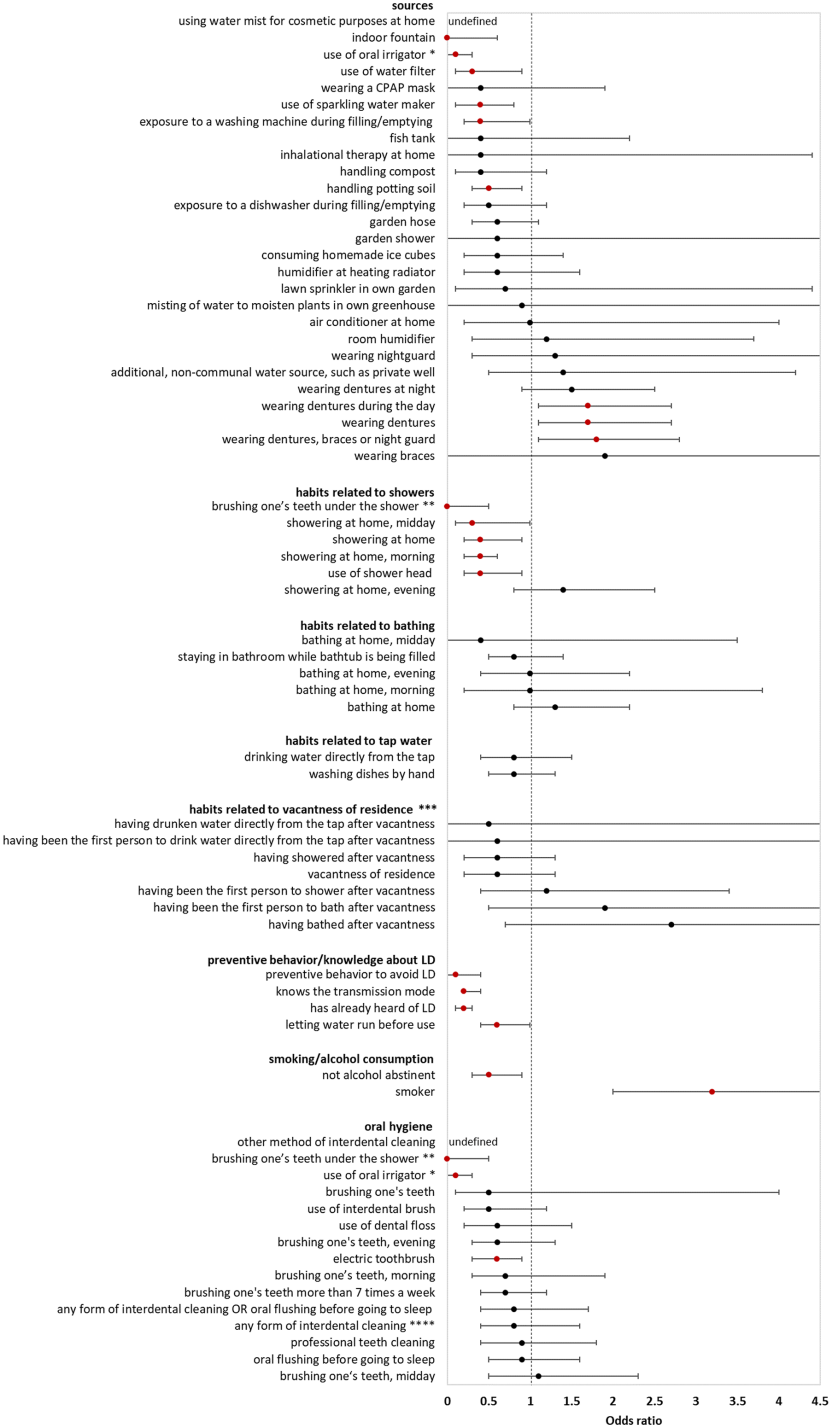
Results of bivariate analyses of potential sources, behavior, knowledge, and oral hygiene of cases who likely or presumably acquired Legionnaires’ disease at home (AHALD) with controls. Significant variables are shown in red. LD, Legionnaires' disease, * “use of oral irrigator” is shown in two FIGURE 1 (Continued)categories: “oral hygiene” and “sources”, ** “brushing one’s teeth under the shower” is shown in two categories: “oral hygiene” and “habits related to showers”, *** vacantness of residence = before using the water sources, the residence was unoccupied for more than 24 h, **** “any form of interdental cleaning” is an overarching variable, sub-questions included “use of interdental brush,” “use of dental floss,” “professional teeth cleaning” and “other method of interdental cleaning”.

**Figure 2:**
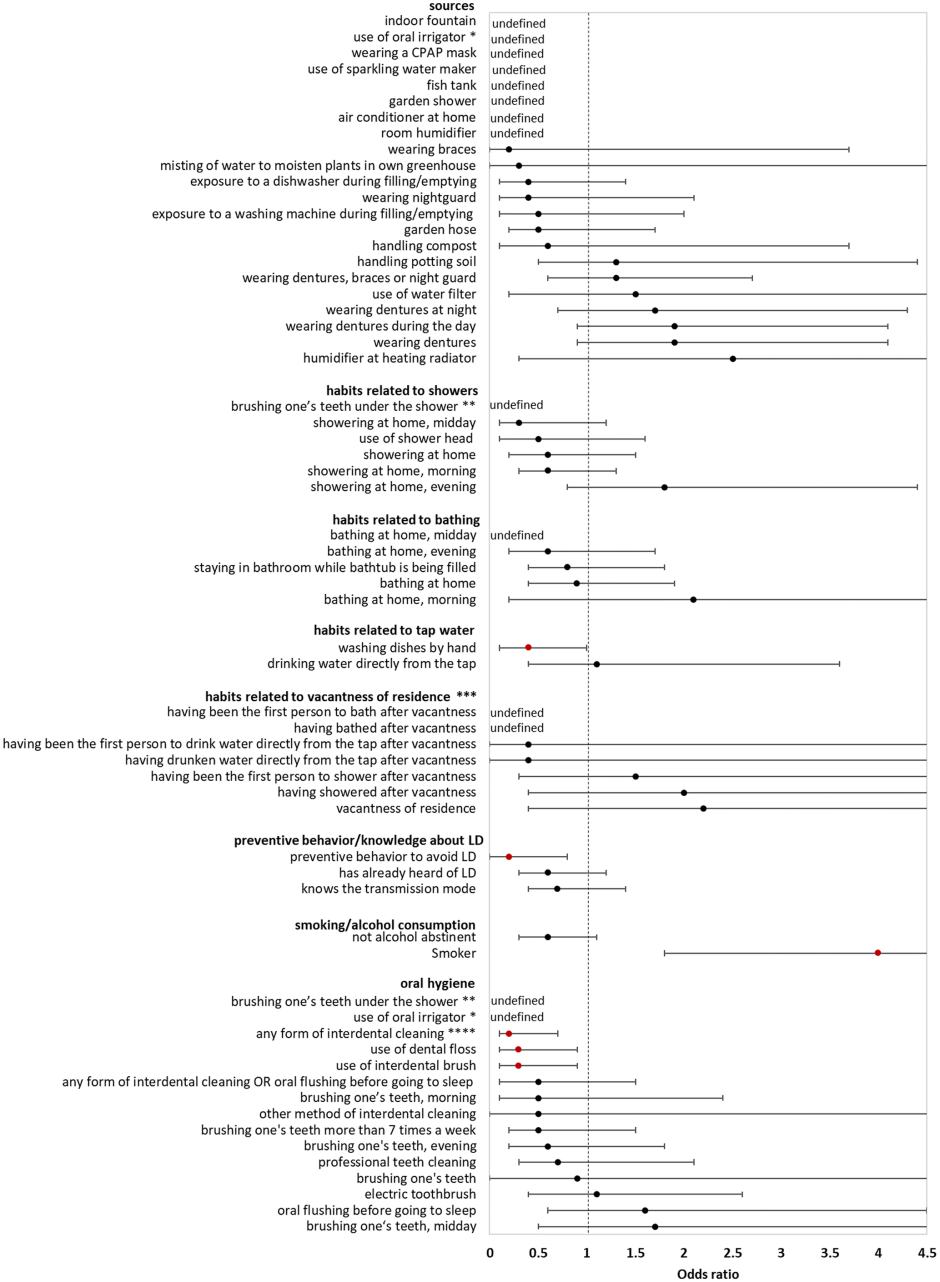
Results of bivariate analyses of potential sources, behavior, knowledge and oral hygiene of cases who likely or presumably acquired Legionnaires’ disease at home (AHALD) with household members (AHALD-HHM). Some variables are missing because they were not asked to AHALD-HHM or it was in retrospect not clear if the source was a household or external source. Significant variables are shown in red. LD, Legionnaires' disease,* “use of oral irrigator” is shown in two categories: “oral hygiene” and “sources”, ** “brushing one’s teeth under the shower” is shown in two categories: “oral hygiene” and “habits related to showers”, *** vacantness of residence = before using the water sources, the residence was unoccupied for more than 24 h, **** “any form of interdental cleaning” is an overarching variable, sub-questions included “use of interdental brush,” “use of dental floss,” “professional teeth cleaning” and “other method of interdental cleaning”.

In the **bivariate analysis of sources** (using **controls** for comparison) AHALD were not associated with infection sources potentially producing infectious aerosol. The only source significantly associated with AHALD was wearing dentures (compared with controls; OR = 1.7, 95% CI = 1.1–2.7, value of *p* = 0.02). Other sources, such as exposure to a humidifier or a garden shower, handling compost or potting soil had an OR below 1 (Figure 1). The OR for handling potting soil, exposure to indoor fountain, washing machine during filling/emptying, use of oral irrigation, use of a water filter and use of sparkling water maker were even significantly below 1 (Figure 1). The OR were similar when cases with AHALD were compared with **AHALD-HHM**. The association with wearing dentures was similar in magnitude (compared with the analysis using controls), however, the association was not significant due to insufficient statistical power (Figure 2). Also, the associations of most other sources had an OR below 1. Only few variables contradicted the OR of the analysis with controls because they lay on the opposite side of 1, such as handling potting soil or use of water filter. However, none of these were statistically significant.

**Behavior/habits related to sources** (using **controls** for comparison) yielded an OR for “showering at home” which was significantly lower than 1 (OR = 0.4, 95% CI = 0.2–0.9, value of *p* = 0.01; Figure 1) as well as a negative association for “brushing one’s teeth under the shower” (OR = 0, 95% CI = 0–0.5, value of *p* = 0.003). Variables regarding habits related to bathing, use of tap water, vacantness of residence or showering/bathing after vacantness were all not significant (Figure 1). When cases with AHALD were compared to **AHALD-HHM** “showering at home” had an OR of 0.6 (95% CI = 0.2–1.5, value of *p* = 0.25; Figure 2). The OR for “brushing one’s teeth under the shower” remained undefined because neither a case nor any AHALD-HHM indicated to have done that. Similar to the comparison with the controls variables regarding habits related to bathing, use of tap water, vacantness of residence or showering/bathing after vacantness were all not significant, with one exception: washing dishes by hand was associated with an OR significantly below 1 (Figure 2).

Variables covering **knowledge** of LD and preventive behavior (using **controls** for comparison), such as letting water run before using it, were all significantly negatively associated with AHALD. For the comparison with **AHALD-HHM** ORs went in the same direction, however, only “preventive behavior to avoid LD” was significantly negatively associated with AHALD.

Regarding **smoking and alcohol consumption,** being a smoker was strongly significantly associated with a positive OR in both comparison groups, while “not being alcohol abstinent” was negatively associated, upon the comparison with controls significantly so (Figure 1).

Three variables related to **oral hygiene** (using **controls** for comparison) were significantly negatively associated with AHALD: “brushing one’s teeth under the shower,” use of oral irrigator and use of electric toothbrush. For the comparison with **AHALD-HHM** the overarching variable “any form of interdental cleaning” and the more specific variables use of dental floss and use of interdental brush were significantly negatively associated with AHALD. In both comparison groups several other variables had an OR below 1, but were not significant.

All variables are also listed in Supplementary Table S1 in the Appendix.

In the next step we separated cases, controls and AHALD-HHM into two groups: those who said they were wearing dentures and those who said they did not.

### 3.3. Analysis of variables related to wearing dentures

Table 3 shows results of the bivariate analysis of variables related to wearing dentures (using **controls** for comparison). While we asked for different methods of cleaning dentures separately we also created a variable that combined if the person “usually” cleans the dentures with any of two methods (that is, not necessarily at the same point in time): (i) brushing dentures with tap water and tooth paste and (ii) letting them soak in disinfecting solution. This variable combined from two different queried methods (“brushing dentures with tap water and tooth paste, and letting it soak in disinfecting solution”) showed a significant OR below 1 (OR = 0.35, 95% CI = 0.13–0.88, value of *p* = 0.02; Table 3). In addition, “not being alcohol abstinent” was significantly negatively associated (OR = 0.41, 95% CI = 0.19–0.87, value of *p* = 0.01). The OR for consumption of alcohol declined increasingly with increasing alcohol consumption as determined by AUDIT-C (reference = no alcohol consumption; OR for moderate consumption = 0.46, OR for risk consumption = 0.17, value of p for trend = 0.006). Bivariate analysis of cases with AHALD with **AHALD-HHM** yielded also mostly negative OR, however, cell counts were small, and frequently 0, so no OR was statistically significant (Table 3). Therefore, we did not perform multivariable analysis for the comparison of cases with AHALD and AHALD-HHM.

**Table 3:** Results of bivariate analyses of cases with Legionnaires’ disease analyzing variables related to the cleaning of **dentures** as well as to consumption of alcohol; restricted to cases who likely or presumably acquired Legionnaires’ disease at home (AHALD) and who indicated to wear dentures, **(A)** in comparison to controls who indicated to wear dentures, and **(B)** in comparison with household members (AHALD-HHM) who indicated to wear dentures.

	Cases with AHALD			Comparison group: controls						Comparison group: AHALD-HHM					
	*N*	*n*	%	*N*	*n*	%	OR	95% CI	*p*	*N*	*n*	%	OR	95% CI	*p*
**Not alcohol abstinent**	58	26	44.8	75	50	66.7	0.41	0.19–0.87	**0.01**	16	9	56.3	0.63	0.17–2.22	0.43
**Brushing dentures with tap water and tooth paste, AND letting it soak in disinfecting solution**	46	9	19.6	73	30	41.1	0.35	0.13–0.88	**0.02**	12	0	0.0	und.	0.70–und.	0.18
Brushing dentures with tap water and tooth paste	47	34	72.3	73	60	82.2	0.57	0.22–1.50	0.20	12	9	75.0	0.87	0.13–4.28	0.85
Letting dentures soak in disinfecting solution	46	20	43.5	73	39	53.4	0.67	0.30–1.50	0.29	12	2	16.7	3.85	0.69–39.18	0.11
Letting dentures soak in tap water	57	9	15.8	75	8	10.7	1.57	0.50–5.03	0.38	16	0	0.0	und.	0.74–und.	0.19
Brushing dentures with cleaning foam	46	3	6.5	73	8	11.0	0.57	0.09–2.54	0.53	12	0	0.0	und.	und.	und.
Dentures, any soaking liquid	49	26	53.1	71	41	57.8	0.83	0.37–1.84	0.61	12	3	25.0	3.39	0.72–21.7	0.08
Wearing dentures at night	58	37	63.8	75	49	65.3	0.93	0.43–2.04	0.85	16	10	62.5	1.06	0.27–3.77	0.92
Any other form of cleaning dentures	46	3	6.5	73	4	5.5	1.20	0.17–7.48	1	12	0	0.0	und.	und.	und.
Brushing dentures with tap water only	47	2	4.3	73	3	4.1	1.04	0.08–9.42	1	12	0	0.0	und.	und.	und.
Flushing dentures with tap water only	47	0	0.0	73	1	1.4	0.00	und.	und.	12	1	8.35	und.	und.	und.
Wearing dentures during the days	58	58	100	75	75	100	und.	und.	und.	16	16	100	und.	und.	und.

The table is ordered by value of *p* in the comparison cases with controls; LeTriWa study, Berlin, Germany, 2016–2020. OR, Odds ratio; CI, confidence interval; *p*, value of *p*; und., undefined; variables significant in at least one comparison group are shown in bold.

In the multivariable analysis for the comparison of cases with AHALD with **controls** the variable “any alcohol consumption” (category 2 or 3 in Audit-C) and the variable capturing the cleaning routine of dentures (“brushing dentures with tap water and tooth paste, as well as letting dentures soak in disinfecting solution”) have similar OR and remained significant when adjusting to age group, sex, educational level and smoking (Table 4).

**Table 4:** Multivariable logistic regression model for cases with Legionnaires’ disease analyzing variables related to the cleaning of **dentures**; restricted to cases who likely or presumably acquired Legionnaires’ disease at home (**AHALD**) and who indicated to wear dentures, and **controls** who indicated to wear dentures; LeTriWa-study, Berlin, Germany, 2016–2020.

		Odds radio	95% CI	value of *p*
**Brushing dentures with tap water and tooth paste, AND letting it soak in disinfecting solution**		0.33	0.13–0.83	**0.02**
**Not alcohol abstinent**		0.36	0.15–0.84	**0.02**
>74 years old		0.81	0.33–2.01	0.65
Male sex		1.80	0.76–4.26	0.18
**High school or university graduation**		0.26	0.09–0.79	**0.02**
Smoker		1.99	0.79–5.00	0.15

CI, confidence interval; significant variables are shown in bold.

### 3.4. Analyses of general oral hygiene variables among persons not wearing dentures

In the comparison of cases with AHALD with **controls** many variables coding for behavior or habits related to oral hygiene had an OR below 1 (Table 5). Statistically significant were “use of oral irrigator,” “use of interdental brush,” “brushing one’s teeth >7 times a week” and “brushing one’s teeth in the morning.” Five controls used even both an oral irrigator and interdental brush and brushed their teeth >7 times a week, but no case with AHALD practiced all three methods. In the multivariable analysis we combined these three variables and calculated the OR when any of these three habits were practiced in relation to none of the three. The OR was 0.32 (95% CI = 0.10–1.04, value of *p* = 0.06; Table 6) adjusted for “no alcohol abstinence,” age group, sex, educational level and smoking.

**Table 5:** Results of bivariate analyses of cases with Legionnaires’ disease analyzing variables related to **oral hygiene** as well as consumption of alcohol; restricted to cases who likely or presumably acquired Legionnaires’ disease at home (AHALD) and who indicated to wear no dentures, **(A)** in comparison to controls who indicated to wear no dentures, and **(B)** in comparison to household members (AHALD-HHM) who indicated to wear no dentures.

	Cases with AHALD			Comparison group: controls						Comparison group: AHALD-HHM					
	*N*	*n*	%	*N*	*n*	%	OR	95% CI	*p*	*N*	*n*	%	OR	95% CI	*p*
**Use of oral irrigator**	63	0	0.0	138	25	18.1	0.00	0.00–0.28	**<0.001**	24	0	0.0	und.	und.	und.
**Use of interdental brush**	33	4	12.1	77	26	33.8	0.27	0.06–0.90	**0.02**	18	8	44.4	0.17	0.03–0.84	**0.02**
**Brushing one’s teeth more than 7 times a week**	49	34	69.4	107	90	84.1	0.43	0.18–1.04	**0.04**	25	21	84.0	0.43	0.09–1.62	0.17
**Brushing one’s teeth, morning**	49	42	85.7	107	102	95.3	0.29	0.07–1.16	**0.04**	25	24	96.0	0.25	0.01–2.17	0.18
Any form of interdental cleaning* OR oral flushing before going to sleep	38	24	63.2	86	68	79.1	0.45	0.18–1.16	0.06	20	17	85.0	0.30	0.05–1.36	0.08
Brushing one’s teeth, evening	49	38	77.6	107	94	87.9	0.48	0.18–1.30	0.10	25	22	88.0	0.47	0.08–2.07	0.28
Brushing one’s teeth	49	47	95.9	107	106	99.1	0.22	0.00–4.40	0.18	25	25	100	0.00	0.00–3.80	0.32
Any form of interdental cleaning*	35	19	54.3	78	51	65.4	0.63	0.26–1.54	0.26	18	15	83.3	0.24	0.04–1.09	**0.04**
Electric toothbrush	61	27	44.3	135	71	52.6	0.72	0.37–1.37	0.28	24	9	37.5	1.32	0.46–3.99	0.57
Other method of interdental cleaning	33	1	3.0	77	0	0.0	und.	und.	und.	18	1	5.6	0.53	0.01–44.13	1
Use of dental floss	33	8	24.2	77	25	32.5	0.67	0.23–1.81	0.39	18	8	44.4	0.40	0.10–1.63	0.14
Oral flushing before going to sleep	49	12	24.5	106	33	31.1	0.72	0.30–1.63	0.40	25	7	28.0	0.83	0.25–2.96	0.74
Not alcohol abstinent	63	45	71.4	138	106	76.8	0.75	0.37–1.59	0.41	33	28	84.9	0.45	0.12–1.45	0.14
Professional teeth cleaning	35	14	40.0	78	36	46.2	0.78	0.32–1.88	0.54	18	7	38.9	1.05	0.28–4.02	0.94
Brushing one‘s teeth, midday	49	6	12.2	105	15	14.3	0.84	0.25–2.49	0.73	25	2	8.0	1.60	0.26–17.39	0.71

The table is ordered by value of *p* in the comparison cases with controls; LeTriWa study, Berlin, Germany, 2016–2020. OR, Odds ratio; CI, confidence interval; *p*, value of *p*; und., undefined; variables significant in at least one comparison group are shown in bold. * “Any form of interdental cleaning” is an overarching variable integrating the variables “Use of interdental brush,” “Use of dental floss,” “Professional teeth cleaning” and “Other method of interdental cleaning”.

**Table 6:** Multivariable logistic regression model for cases with Legionnaires’ disease analyzing variables related to **oral hygiene**; restricted to cases who likely or presumably acquired Legionnaires’ disease at home (**AHALD**) and who indicated to wear no dentures, in comparison to **controls** and who indicated to wear no dentures, and in comparison to household members (**AHALD-HHM**) who indicated to wear no dentures; LeTriWa study, Berlin, Germany, 2016–2020.

		Odds ratio	95% CI	value of *p*
**Comparison group: controls**				
Habit related to oral hygiene				
Brushing one’s teeth ≤7 times a week AND no use of oral irrigator AND no use of interdental brush		reference		
Brushing one’s teeth >7 times a week OR use of oral irrigator OR use of interdental brush		0.32	0.10–1.04	0.06
Not alcohol abstinent		0.41	0.16–1.07	0.07
**>74 years old**		4.53	1.72–11.94	**0.002**
Male sex		1.32	0.55–3.21	0.54
High school or university graduation		0.64	0.28–1.44	0.28
**Smoker**		6.11	2.41–15.49	**<0.001**
**Comparison group: AHALD-HHM**				
Use of interdental brush		0.18	0.03–1.20	0.08
Not alcohol abstinent		0.35	0.06–1.91	0.23
>74 years old		4.37	0.75–25.41	0.10
Male sex		2.90	0.65–12.92	0.16
High school or university graduation		0.85	0.20–3.57	0.83
Smoker		3.26	0.63–16.91	0.16

CI, confidence interval; significant variables are shown in bold.

In the bivariate analysis of cases with AHALD comparing with **AHALD-HHM** of behaviors/habits related to oral hygiene the overarching variable “any form of interdental cleaning” (with the sub-questions “use of interdental brush,” “use of dental floss,” “professional teeth cleaning” or “other method of interdental cleaning”) was statistically significantly negatively associated (OR = 0.24, 95% CI = 0.04–1.09, value of *p* = 0.04; Table 5), which was also the case for “use of interdental brush” (OR = 0.17, 95% CI = 0.03–0.84, value of *p* = 0.02; Table 5). As “use of interdental brush” was part of the overarching variable “any form of interdental cleaning” we performed multivariable analyses with the more specific variable “use of interdental brush.” In that model the variable “use of interdental brush” had an OR of 0.18, but was not significant when we adjusted for “no alcohol abstinence,” age group, sex, educational level and smoking (Table 6).

## 4. Discussion

Using data from the LeTriWa study in Berlin we showed that household samples of cases with AHALD more frequently contained MAb 3/1-positive strains than those of controls. Our epidemiological analysis of the questionnaire variables related to exposures to many possible infection sources in the household did not demonstrate that any infection source, including shower, bathing and tap water were significantly positively associated, even when analyzed via behavioral variables. The one notable exception was “wearing dentures” (in the comparison of cases with AHALD and controls). After separating cases with AHALD, controls and AHALD-HHM in those wearing/not wearing dentures we identified surprisingly strong and consistent preventive effects for behaviors/habits related to oral hygiene: (1) both for the denture-wearing and not denture-wearing groups, (2) for both comparison groups (controls and AHALD-HHM), (3) consistently adjusted for “no alcohol abstinence,” age group, sex, educational level and smoking, and (4) with some additional microbiological support (detection of *Legionella* bacteria in a denture scratch sample).

The fact that household water and biofilm samples from bathroom faucets and showers contained significantly more frequently MAb 3/1-positive strains confirms the crucial role of residential water in transmission of legionellae. However, in itself it does not tell us where and how cases acquired LD, only that these strains may be introduced into the household via residential drinking water. For example, water from the tap, the shower or the bathtub could have been used for other sources, such as a watering can, humidifier or to soak dentures. Indeed, we identified not only *Legionella* spp. bacteria, but also MAb 3/1-positive strains in samples of, e.g., water containers, a humidifier or potting soil. As these sources were not sampled systematically in case and control households we used the information from the questionnaire to analyzed the exposure to potential sources comparing cases with AHALD with controls, as well as cases with AHALD and AHALD-HHM.

Commonly showers are considered the major source for transmission of LD at home resulting for example in the recommendation to stop using showers when very high *Legionella* concentrations are found in building water systems [DVGW (Deutscher Verein des Gas- und Wasserfachs), 2004]. While showers are often assumed to be the main source of infection in indoor settings, we found only two outbreak investigations of (nosocomial) cases of LD that identified showers as a risk factor for LD (Breiman et al., 1990; Silk et al., 2013). In our study, neither “showering at home” nor any other behavioral variable related to shower use was significantly positively associated with AHALD, neither in the comparison of cases with controls nor in the comparison of cases with AHALD-HHM. In the contrary, several of these variables had an OR significantly below 1, in addition the OR in both control groups were quite similar. We obtained similar results from other potential sources, such as habits related to bathing, to tap water, water filter or handling potting soil. It is therefore unlikely that any of these exposures contributes a substantial number of cases with AHALD to the burden of CALD – notwithstanding the possibility that individual cases may acquire their infection through one of these sources. However, if conversely any of these have a truly preventive role this would need to be explored further. Only wearing dentures was significantly positively associated and had the same OR in both comparisons (cases with AHALD versus controls, and cases with AHALD versus AHALD-HHM). We reported a similar result in a previous paper when the study was not completed yet (Buchholz et al., 2020).

Controls indicated significantly more frequently than cases with AHALD that they were trying to avoid LD through their behavior, that they knew the transmission mode, had already heard of LD before and let water run before its use (Figure 1). Comparison in the AHALD-HHM group yielded also ORs below 1 (Figure 2). In how far these differences represent a behavior that truly reduces the risk of acquiring LD is unknown, as answers to these questions may be biased. For example, out of a defensive reflex cases may have been inclined to answer more frequently that they did not know the transmission mode for the acquisition of LD.

Similar to the results in our study smoking has been shown previously to be strongly associated with LD (Storch et al., 1979; Straus et al., 1996; Den Boer et al., 2006). Calculating the population attributable risk approximately a third of all cases of LD would not happen if no one smoked anymore. We expected also that consumption of alcohol would be a risk factor for LD. However, contrary to this assumption, the consumption of alcohol seemed to have a protective effect (Figure 1). While at first sight this finding came as a surprise we found that in the named three studies results for alcohol were quite mixed (Storch et al., 1979; Straus et al., 1996; Den Boer et al., 2006). We hypothesized that alcohol may have a “preventive” effect because it might kill bacteria in the oral cavity and explored its role together with the other oral hygiene variables.

The potential role of oral hygiene as a preventive factor for the acquisition of LD was unexpected. For example, we included the variable “brushing one’s teeth under the shower” because we suspected this might be a behavior putting persons at risk by potentiating the effect of having a shower. Moreover, only because dentures were known to harbor biofilm (Schmutzler et al., 2021) we included “wearing dentures” as variable in the questionnaire. Because it emerged as a risk factor in interim analyses we added most of the variables related to oral hygiene after the field work had started already. After dividing the groups into those who indicated to wear dentures and those who did not we found that the preventive effect of alcohol consumption is mainly apparent in the denture wearing group. In the multivariable logistic regression analysis restricted to **denture wearing** cases and denture wearing controls the effect of (drinking) alcohol was evaluated together with the variable “brushing dentures with tap water and tooth paste, and letting it soak in disinfecting solution” and it showed that both exposures had a significantly “preventive” effect. As we identified these associations already in the course of the study we began to take also samples from dentures of cases. In one scratch sample we found *Legionella* bacteria, as well as in a sample of another case-patient with AHALD where a biofilm sample of the denture was diluted in the water used for soaking the denture. In the multivariable analysis of the comparison of cases with AHALD versus controls who indicated to wear no dentures we found that any of three habits related to **oral hygiene** (brushing one’s teeth >7 times a week, use of oral irrigator, use of interdental brush) lowered the association with LD. In the comparison with AHALD-HHM use of interdental brush was negatively associated with LD, albeit in the final multivariable model not significantly anymore. Taking these results together we developed the hypothesis that there is a possibility that *Legionella* live for example on biofilm or in plaques in the oral cavity and that these might be inhaled and thus lead to pneumonia.

We believe that we have gathered a substantial amount of converging evidence. **First**, the only variable among the potential sources that was significantly associated was wearing dentures, possibly via biofilm forming on dentures (Schmalz and Cieplik, 2021; Schmutzler et al., 2021). **Second**, we found oral hygiene variables to have a significantly negative OR (“preventive”) in both subgroups: those with wearing dentures and those without. To our knowledge no other study has investigated oral hygiene as a preventive factor for LD. **Third**, although the effects in the comparison of cases with AHALD with AHALD-HHM were not as strong as those in the comparison of cases with AHALD with controls they went into the same direction, and it was probably just a matter of lower power that they were not significant, too. Thus, we identified these effects in both comparison groups: the controls AND AHALD-HHM. **Fourth**, adjustment for other relevant variables, namely “no alcohol abstinence,” age group, sex, education and smoking that could well be conceived as potential confounders in this context strengthens the interpretation that the found associations are true. **Fifth,** the fact that we found at least in two instances *Legionella* via PCR on dentures (or water used to soak dentures) of cases with AHALD, backs our findings on microbiological grounds. As dentures may be a huge reservoir of bacteria (Fujinami et al., 2021) and also dental plaques can contain millions of bacteria in just one cubic millimeter (Thoden van Velzen et al., 1984) with an estimated 19,000 phylotypes (Keijser et al., 2008) it is biologically plausible that *Legionella* may be present in dentures and/or dental plaque. To our knowledge only few studies have looked for *Legionella* in oral biofilm and one study that did has identified *Legionella* DNA via PCR on dental plaques (Tesauro et al., 2018). And **sixth,** both the increased risk of aspiration pneumonia (in general, i.e., independently of the agent) with inadequate oral care (Quagliarello et al., 2005) as well as the preventive effect of good oral and denture hygiene on the risk of pneumonia in general has already been shown in several studies (Yoneyama et al., 1999; Adachi et al., 2002; El-Solh, 2011; Sjogren et al., 2016; Son et al., 2020). One possible pathogenetic mechanism is the (micro-)aspiration of oral bacteria into the lower respiratory tract (Scannapieco and Ho, 2001). The finding of Iinuma et al. that the risk of pneumonia can be reduced if dentures are not worn at night suggests that aspiration occurs preferentially during night rest (Iinuma et al., 2015).

If *Legionella* in the oral cavity would truly be a source of LD simple additional measures, related to improved oral hygiene, could be recommended to prevent Legionnaires’ disease. Thus, further epidemiological and microbiological studies are needed to scrutinize our results.

We would like to mention the following limitations. First, we did not reach the intended 2:1 ratio of controls-to-cases, in addition not all HHM agreed to participate, both of which limited our power to detect differences. Second, we considered cooling towers as potential sources only in a limited way, that is, when the residences of several cases were in relatively short distance to each other. We would have considered cooling towers as a potential source, of course, had there been an apparent outbreak of Legionnaires’ disease. And we also considered cooling towers as an infection source when there was a professional exposure. In no case we found suggestive evidence that supported the role of a cooling tower or evaporative condenser for the acquisition of Legionnaires’ disease. Third, not all potential infection sources in the household were investigated. For example, aerosol exposure to contaminated siphons or toilets cannot be assessed with the use of questionnaires and need thorough controlled and standardized microbiological sampling. Fourth, the bulk of the presented evidence for the potential preventive effect of oral hygiene derives from just this single case–control study and is by and large epidemiological in nature. Further studies are needed both with epidemiological as well as microbiological focus.

## 5. Conclusion

In conclusion, and as response to the initial questions AHALD was not associated with having a shower, having a bath or many other behavioral factors or infection sources in the household. The only source or “risk behavior” with evidence from this study is to wear dentures. Smoking was confirmed as strong risk factor for LD. Several oral hygiene variables both among persons wearing dentures and those who do not may have a “preventive” effect. If confirmed these results could have substantial consequences for public health practice. Further epidemiological and microbiological studies should be conducted to scrutinize our findings.

## Data availability statement

The datasets presented in this article are not readily available because the data in this study are restricted by the Federal Commissioner for Data Protection and Freedom of Information, and the Ethics Committee of the Charité Medical University of Berlin. Requests to access the datasets should be directed to datenschutz@rki.de.

## Ethics statement

The studies involving human participants were reviewed and approved by the Ethics Committee of the Charité (Medical University of Berlin, Campus Charité Mitte, Charitéplatz 1, 10117 Berlin), application number EA1/303/15. The patients/participants provided their written informed consent to participate in this study.

## Author contributions

UB and A-SL conceived and designed the study and performed the statistical analysis. FR, ML, HJ, and UB have taken the samples. BS, CL, and MP were involved in the processing of the samples. UB wrote the first draft of the manuscript. A-SL, FR, ML, HJ, CL, MP, BS, A-RG, and KL reviewed and edited the manuscript. All authors contributed to the article and approved the submitted version.

## Funding

The LeTriWa study was financed by the German Federal Ministry of Health (grant number ZMVI5-2515-FSB-759).

## Conflict of interest

The authors declare that the research was conducted in the absence of any commercial or financial relationships that could be construed as a potential conflict of interest.

## Publisher’s note

All claims expressed in this article are solely those of the authors and do not necessarily represent those of their affiliated organizations, or those of the publisher, the editors and the reviewers. Any product that may be evaluated in this article, or claim that may be made by its manufacturer, is not guaranteed or endorsed by the publisher.

## Abbreviations

AHALD: at-home-acquired Legionnaires’ disease
CALD: community-acquired Legionnaires’ disease
HHM: household member
LD: Legionnaires’ disease
LeTriWa: Legionellen im Trinkwasser (*Legionella* in drinking water) (acronym for the LeTriWa study)
MAb: monoclonal antibody
