# Supplementary material for: Could oral hygiene prevent cases of at-home-acquired Legionnaires’ disease? – Results of a comprehensive case–control study on infection sources, risk, and protective behaviors
Source: Front Microbiol. 2023 Jun 15;14:1199572. doi: 10.3389/fmicb.2023.1199572 (PMC10311500; doi:10.3389/fmicb.2023.1199572)
Supplement: Supplementary file 1 [file Data_Sheet_1.docx]

Supplementary Material

Could oral hygiene prevent cases of at-home-acquired Legionnaires' disease? – results of a comprehensive case-control study on infection sources, risk and protective behaviors

Ann-Sophie Lehfeld*, Franziska Reber, Marina M. Lewandowsky, Heiko J. Jahn, Christian Lück, Markus Petzold, Benedikt Schaefer, Anna-Rachel Germelmann, Katrin Lorenz, Udo Buchholz

*** Correspondence:** Ann-Sophie Lehfeld: [LehfeldA@rki.de](mailto:LehfeldA@rki.de)


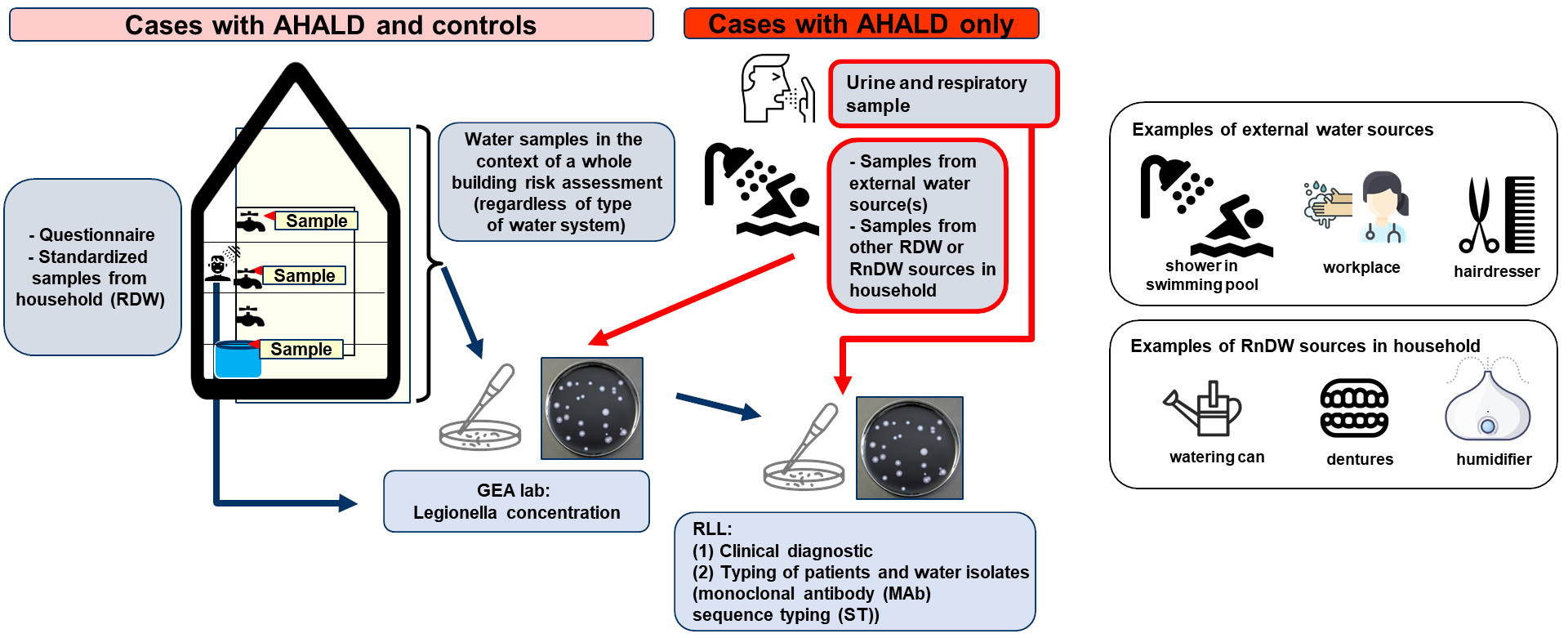


**Supplementary Figure S1.** Schematic overview of data collection in the study.
AHALD = at-home-acquired Legionnaires' disease; RDW = residential drinking water; RnDW = residential non-drinking water; GEA = German Environment Agency; RLL = reference laboratory for Legionella; MAb = monoclonal antibody; ST = sequence type.

**Supplementary Table 1.** Results of bivariate analyses of potential sources, behavior, knowledge and oral hygiene of cases who likely or presumably acquired Legionnaires' disease at home (AHALD) with controls, and household members (AHALD-HHM), respectively. Some variables are missing because they were not asked to AHALD-HHM or it was in retrospect not clear if the source was a household or external source. Variables significant in at least one comparison group are shown in bold; LeTriWa study, Berlin, Germany, 2016–2020.

|  |  | **Cases with AHALD** | | |  | **Comparison group: Controls** | | | | | |  | **Comparison group: HHM-AHALD** | | | | | |
| --- | --- | --- | --- | --- | --- | --- | --- | --- | --- | --- | --- | --- | --- | --- | --- | --- | --- | --- |
| **Category** | | **N** | **n** | **%** |  | **N** | **n** | **%** | **OR** | **95% CI** | **p** |  | **N** | **n** | **%** | **OR** | **95% CI** | **p** |
| **sources** | |  |  |  |  |  |  |  |  |  |  |  |  |  |  |  |  |  |
|  | using water mist for cosmetic purposes at home | 122 | 0 | 0 |  | 214 | 1 | 0 | und. | und. | 1 |  |  |  |  |  |  |  |
|  | **indoor fountain** | 124 | 0 | 0 |  | 214 | 10 | 5 | 0 | 0.0–0.6 | **0.02** |  | 36 | 0 | 0 | und. | und. | und. |
|  | **use of oral irrigator *** | 123 | 1 | 1 |  | 214 | 29 | 14 | 0.1 | 0.0–0.3 | **< 0.001** |  | 36 | 0 | 0 | und. | und. | und. |
|  | **use of water filter** | 123 | 5 | 4 |  | 214 | 25 | 12 | 0.3 | 0.1–0.9 | **0.02** |  | 36 | 1 | 3 | 1.5 | 0.2–72 | 1 |
|  | wearing a CPAP mask | 124 | 2 | 2 |  | 214 | 9 | 4 | 0.4 | 0.0–1.9 | 0.34 |  | 36 | 0 | 0 | und. | und. | und. |
|  | **use of sparkling water maker** | 124 | 8 | 6 |  | 214 | 34 | 16 | 0.4 | 0.1–0.8 | **0.01** |  | 36 | 0 | 0 | und. | und. | und. |
|  | **exposure to a washing machine during filling/emptying** | 39 | 26 | 67 |  | 99 | 82 | 83 | 0.4 | 0.2–1.0 | **0.04** |  | 20 | 16 | 80 | 0.5 | 0.1–2.0 | 0.28 |
|  | fish tank | 124 | 2 | 2 |  | 214 | 8 | 4 | 0.4 | 0.0–2.2 | 0.34 |  | 36 | 0 | 0 | und. | und. | und. |
|  | inhalational therapy at home | 123 | 1 | 1 |  | 214 | 4 | 2 | 0.4 | 0.0–4.4 | 0.66 |  |  |  |  |  |  |  |
|  | handling compost | 124 | 6 | 5 |  | 214 | 22 | 10 | 0.4 | 0.1–1.2 | 0.08 |  | 36 | 3 | 8 | 0.6 | 0.1–3.7 | 0.42 |
|  | **handling potting soil** | 123 | 26 | 21 |  | 212 | 72 | 34 | 0.5 | 0.3–0.9 | **0.01** |  | 36 | 6 | 17 | 1.3 | 0.5–4.4 | 0.56 |
|  | exposure to a dishwasher during filling/emptying | 41 | 22 | 54 |  | 99 | 68 | 69 | 0.5 | 0.2–1.2 | 0.09 |  | 20 | 15 | 75 | 0.4 | 0.1–1.4 | 0.11 |
|  | garden hose | 123 | 14 | 11 |  | 214 | 40 | 19 | 0.6 | 0.3–1.1 | 0.08 |  | 36 | 7 | 19 | 0.5 | 0.2–1.7 | 0.26 |
|  | garden shower | 123 | 1 | 1 |  | 214 | 3 | 1 | 0.6 | 0.0–7.3 | 1 |  | 36 | 0 | 0 | und. | und. | und. |
|  | consuming homemade ice cubes | 121 | 8 | 7 |  | 211 | 23 | 11 | 0.6 | 0.2–1.4 | 0.20 |  |  |  |  |  |  |  |
|  | humidifier at heating radiator | 122 | 8 | 7 |  | 213 | 21 | 10 | 0.6 | 0.2–1.6 | 0.30 |  | 36 | 1 | 3 | 2.5 | 0.3–112 | 0.69 |
|  | lawn sprinkler in own garden | 119 | 2 | 2 |  | 212 | 5 | 2 | 0.7 | 0.1–4.4 | 1 |  |  |  |  |  |  |  |
|  | misting of water to moisten plants in own greenhouse | 122 | 1 | 1 |  | 213 | 2 | 1 | 0.9 | 0.0–17 | 1 |  | 36 | 1 | 3 | 0.3 | 0.0–23 | 0.41 |
|  | air conditioner at home | 123 | 4 | 3 |  | 214 | 7 | 3 | 1 | 0.2–4.0 | 1 |  | 36 | 0 | 0 | und. | und. | und. |
|  | room humidifier | 124 | 6 | 5 |  | 213 | 9 | 4 | 1.2 | 0.3–3.7 | 0.79 |  | 36 | 0 | 0 | und. | und. | 0.34 |
|  | wearing nightguard | 96 | 4 | 4 |  | 183 | 6 | 3 | 1.3 | 0.3–5.6 | 0.74 |  | 37 | 4 | 11 | 0.4 | 0.1–2.1 | 0.22 |
|  | additional, non-communal water source, such as private well | 122 | 8 | 7 |  | 215 | 10 | 5 | 1.4 | 0.5–4.2 | 0.46 |  |  |  |  |  |  |  |
|  | wearing dentures at night | 121 | 37 | 31 |  | 213 | 49 | 23 | 1.5 | 0.9–2.5 | 0.13 |  | 49 | 10 | 20 | 1.7 | 0.7–4.3 | 0.18 |
|  | **wearing dentures during the day** | 121 | 58 | 48 |  | 213 | 75 | 35 | 1.7 | 1.1–2.7 | **0.02** |  | 49 | 16 | 33 | 1.9 | 0.9–4.1 | 0.07 |
|  | **wearing dentures** | 121 | 58 | 48 |  | 213 | 75 | 35 | 1.7 | 1.1–2.7 | **0.02** |  | 49 | 16 | 33 | 1.9 | 0.9–4.1 | 0.07 |
|  | **wearing dentures, braces or night guard** | 121 | 62 | 51 |  | 213 | 80 | 38 | 1.8 | 1.1–2.8 | **0.02** |  | 49 | 22 | 45 | 1.3 | 0.6–2.7 | 0.45 |
|  | wearing braces | 96 | 1 | 1 |  | 183 | 1 | 1 | 1.9 | 0.0–151 | 1 |  | 37 | 2 | 5 | 0.2 | 0.0–3.7 | 0.19 |
| **habits related to showers** | |  |  |  |  |  |  |  |  |  |  |  |  |  |  |  |  |  |
|  | **brushing one’s teeth under the shower **** | 123 | 0 | 0 |  | 213 | 13 | 6 | 0 | 0.0–0.5 | **0.003** |  | 58 | 0 | 0 | und. | und. | und. |
|  | **showering at home, midday** | 97 | 4 | 4 |  | 166 | 20 | 12 | 0.3 | 0.1–1.0 | **0.03** |  | 43 | 6 | 14 | 0.3 | 0.1–1.2 | 0.07 |
|  | **showering at home** | 123 | 100 | 81 |  | 214 | 195 | 91 | 0.4 | 0.2–0.9 | **0.01** |  | 59 | 52 | 88 | 0.6 | 0.2–1.5 | 0.25 |
|  | **showering at home, morning** | 97 | 43 | 44 |  | 165 | 114 | 69 | 0.4 | 0.2–0.6 | **< 0.001** |  | 43 | 25 | 58 | 0.6 | 0.3–1.3 | 0.13 |
|  | **use of shower head** | 123 | 107 | 87 |  | 215 | 204 | 95 | 0.4 | 0.2–0.9 | **0.01** |  | 59 | 55 | 93 | 0.5 | 0.1–1.6 | 0.21 |
|  | showering at home, evening | 97 | 37 | 38 |  | 167 | 51 | 31 | 1.4 | 0.8–2.5 | 0.21 |  | 43 | 11 | 26 | 1.8 | 0.8–4.4 | 0.15 |
| **habits related to bathing** | |  |  |  |  |  |  |  |  |  |  |  |  |  |  |  |  |  |
|  | bathing at home, midday | 100 | 1 | 1 |  | 195 | 5 | 3 | 0.4 | 0.0–3.5 | 0.67 |  | 51 | 0 | 0 | und. | und. | und. |
|  | staying in bathroom while bathtub is being filled | 122 | 26 | 21 |  | 213 | 53 | 25 | 0.8 | 0.5–1.4 | 0.46 |  | 59 | 15 | 25 | 0.8 | 0.4–1.8 | 0.54 |
|  | bathing at home, evening | 100 | 11 | 11 |  | 195 | 22 | 11 | 1 | 0.4–2.2 | 0.94 |  | 51 | 9 | 18 | 0.6 | 0.2–1.7 | 0.25 |
|  | bathing at home, morning | 100 | 4 | 4 |  | 195 | 8 | 4 | 1 | 0.2–3.8 | 1 |  | 51 | 1 | 2 | 2.1 | 0.2–105 | 0.66 |
|  | bathing at home | 120 | 35 | 29 |  | 214 | 52 | 24 | 1.3 | 0.8–2.2 | 0.33 |  | 58 | 18 | 31 | 0.9 | 0.4–1.9 | 0.80 |
| **habits related to tap water** | |  |  |  |  |  |  |  |  |  |  |  |  |  |  |  |  |  |
|  | drinking water directly from the tap | 124 | 14 | 11 |  | 214 | 31 | 14 | 0.8 | 0.4–1.5 | 0.41 |  | 57 | 6 | 11 | 1.1 | 0.4–3.6 | 0.88 |
|  | **washing dishes by hand** | 123 | 77 | 63 |  | 213 | 146 | 69 | 0.8 | 0.5–1.3 | 0.27 |  | 36 | 29 | 81 | 0.4 | 0.1–1.0 | **0.04** |
| **habits related to vacantness of residence ***** | |  |  |  |  |  |  |  |  |  |  |  |  |  |  |  |  |  |
|  | having drunken water directly from the tap after vacantness | 115 | 1 | 1 |  | 211 | 4 | 2 | 0.5 | 0.0–4.7 | 0.66 |  | 48 | 1 | 2 | 0.4 | 0.0–33 | 0.50 |
|  | having been the first person to drink water directly from the tap after vacantness | 114 | 1 | 1 |  | 211 | 3 | 1 | 0.6 | 0.0–7.8 | 1 |  | 48 | 1 | 2 | 0.4 | 0.0–33 | 0.51 |
|  | having showered after vacantness | 115 | 9 | 8 |  | 211 | 27 | 13 | 0.6 | 0.2–1.3 | 0.17 |  | 48 | 2 | 4 | 2.0 | 0.4–19 | 0.51 |
|  | vacantness of residence | 115 | 10 | 9 |  | 211 | 30 | 14 | 0.6 | 0.2–1.3 | 0.15 |  | 48 | 2 | 4 | 2.2 | 0.4–21 | 0.51 |
|  | having been the first person to shower after vacantness | 114 | 7 | 6 |  | 205 | 11 | 5 | 1.2 | 0.4–3.4 | 0.77 |  | 48 | 2 | 4 | 1.5 | 0.3–15 | 1 |
|  | having been the first person to bath after vacantness | 114 | 6 | 5 |  | 211 | 6 | 3 | 1.9 | 0.5–7.3 | 0.36 |  | 48 | 0 | 0 | und. | und. | 0.18 |
|  | having bathed after vacantness | 115 | 7 | 6 |  | 211 | 5 | 2 | 2.7 | 0.7–11 | 0.12 |  | 48 | 0 | 0 | und. | und. | 0.11 |
| **preventive behavior/knowledge about LD** | |  |  |  |  |  |  |  |  |  |  |  |  |  |  |  |  |  |
|  | **preventive behavior to avoid LD** | 118 | 4 | 3 |  | 214 | 48 | 22 | 0.1 | 0.0–0.4 | **< 0.001** |  | 59 | 9 | 15 | 0.2 | 0.0–0.8 | **0.01** |
|  | **knows the transmission mode** | 116 | 38 | 33 |  | 214 | 143 | 67 | 0.2 | 0.2–0.4 | **< 0.001** |  | 59 | 24 | 41 | 0.7 | 0.4–1.4 | 0.30 |
|  | **has already heard of LD** | 118 | 65 | 55 |  | 215 | 186 | 87 | 0.2 | 0.1–0.3 | **< 0.001** |  | 59 | 40 | 68 | 0.6 | 0.3–1.2 | 0.11 |
|  | **letting water run before use** | 105 | 59 | 56 |  | 207 | 140 | 68 | 0.6 | 0.4–1.0 | **0.05** |  |  |  |  |  |  |  |
| **smoking/alcohol consumption** | |  |  |  |  |  |  |  |  |  |  |  |  |  |  |  |  |  |
|  | **smoker** | 124 | 59 | 48 |  | 215 | 47 | 22 | 3.2 | 2.0–5.4 | **< 0.001** |  | 59 | 11 | 19 | 4.0 | 1.8–9.2 | **< 0.001** |
|  | **not alcohol abstinent** | 124 | 72 | 58 |  | 217 | 158 | 73 | 0.5 | 0.3–0.9 | **0.01** |  | 59 | 42 | 71 | 0.6 | 0.3–1.1 | 0.09 |
| **oral hygiene** | |  |  |  |  |  |  |  |  |  |  |  |  |  |  |  |  |  |
|  | other method of interdental cleaning | 56 | 2 | 4 |  | 128 | 0 | 0 | und. | und. | 0.09 |  | 27 | 2 | 7 | 0.5 | 0.0–6.8 | 0.59 |
|  | **brushing one’s teeth under the shower **** | 123 | 0 | 0 |  | 213 | 13 | 6 | 0 | 0.0–0.5 | **0.003** |  | 58 | 0 | 0 | und. | und. | und. |
|  | **use of oral irrigator *** | 123 | 1 | 1 |  | 214 | 29 | 14 | 0.1 | 0.0–0.3 | **< 0.001** |  | 36 | 0 | 0 | und. | und. | und. |
|  | brushing one's teeth | 95 | 92 | 97 |  | 179 | 176 | 98 | 0.5 | 0.1–4.0 | 0.43 |  | 37 | 36 | 97 | 0.9 | 0.0–11 | 0.89 |
|  | **use of interdental brush** | 56 | 11 | 20 |  | 128 | 40 | 31 | 0.5 | 0.2–1.2 | 0.11 |  | 27 | 12 | 44 | 0.3 | 0.1–0.9 | **0.02** |
|  | **use of dental floss** | 56 | 9 | 16 |  | 128 | 30 | 23 | 0.6 | 0.2–1.5 | 0.26 |  | 27 | 11 | 41 | 0.3 | 0.1–0.9 | **0.01** |
|  | brushing one's teeth, evening | 94 | 74 | 79 |  | 178 | 152 | 85 | 0.6 | 0.3–1.3 | 0.16 |  | 37 | 32 | 86 | 0.6 | 0.2–1.8 | 0.31 |
|  | **electric toothbrush** | 117 | 37 | 32 |  | 210 | 94 | 45 | 0.6 | 0.3–0.9 | **0.02** |  | 36 | 11 | 31 | 1.1 | 0.4–2.6 | 0.90 |
|  | brushing one’s teeth, morning | 94 | 83 | 88 |  | 178 | 164 | 92 | 0.7 | 0.3–1.9 | 0.44 |  | 37 | 35 | 95 | 0.5 | 0.1–2.4 | 0.35 |
|  | brushing one's teeth more than 7 times a week | 95 | 70 | 74 |  | 179 | 145 | 81 | 0.7 | 0.4–1.2 | 0.16 |  | 37 | 31 | 84 | 0.5 | 0.2–1.5 | 0.22 |
|  | any form of interdental cleaning OR oral flushing before going to sleep | 68 | 47 | 69 |  | 143 | 104 | 73 | 0.8 | 0.4–1.7 | 0.59 |  | 29 | 24 | 83 | 0.5 | 0.1–1.5 | 0.17 |
|  | **any form of interdental cleaning ****** | 58 | 29 | 50 |  | 129 | 70 | 54 | 0.8 | 0.4–1.6 | 0.59 |  | 27 | 22 | 81 | 0.2 | 0.1–0.7 | **0.01** |
|  | professional teeth cleaning | 58 | 19 | 33 |  | 129 | 45 | 35 | 0.9 | 0.4–1.8 | 0.78 |  | 27 | 11 | 41 | 0.7 | 0.3–2.1 | 0.47 |
|  | oral flushing before going to sleep | 95 | 29 | 31 |  | 177 | 58 | 33 | 0.9 | 0.5–1.6 | 0.71 |  | 37 | 8 | 22 | 1.6 | 0.6–4.5 | 0.31 |
|  | brushing one‘s teeth, midday | 94 | 16 | 17 |  | 176 | 27 | 15 | 1.1 | 0.5–2.3 | 0.72 |  | 37 | 4 | 11 | 1.7 | 0.5–7.5 | 0.37 |

OR = Odds ratio, CI = confidence interval, p = p-value, und. = undefined; LD = Legionaries' disease
* “use of oral irrigator” is shown in two categories: “oral hygiene” and “sources”
** “brushing one’s teeth under the shower” is shown in two categories: “oral hygiene” and “habits related to showers”
*** vacantness of residence = before using the water sources, the residence was unoccupied for more than 24 hours
**** “any form of interdental cleaning” is an overarching variable, sub-questions included “use of interdental brush”, “use of dental floss”, “professional teeth cleaning” and “other method of interdental cleaning”
